# Microfluidic Line-Free Mass Sensor Based on an Antibody-Modified Mechanical Resonator

**DOI:** 10.3390/mi9040177

**Published:** 2018-04-12

**Authors:** Masaki Yamaguchi

**Affiliations:** Department of Mechanical Engineering & Robotics, Graduate School of Science & Technology, Shinshu University, 3-15-1 Tokida, Ueda, Nagano 386-8567, Japan; masakiy@shinshu-u.ac.jp; Tel.: +81-268-21-5444

**Keywords:** mass sensor, power supply line, microfluidic, label-free, saliva, cortisol, high-throughput

## Abstract

This research proposes a mass sensor based on mechanical resonance that is free from power supply lines (line-free) and incorporates both microfluidic mechanisms and label-free techniques to improve its sensitivity and reusability. The microfluidic line-free mass sensor comprises a disk-shaped mechanical resonator, a separate piezoelectric element used to excite vibrations in the resonator, and a microfluidic mechanism. Electrical power is used to actuate the piezoelectric element, leaving the resonator free from power lines. The microfluidic mechanism allows for rapid, repeat washings to remove impurities from a sample. The microfluidic line-free mass sensor is designed as a label-free sensor to enable high-throughput by modifying and dissociating an antibody on the resonator. The resonator was fabricated by photolithography and the diameter and thickness were 4 mm and 0.5 mm, respectively. The line-free mass sensor enabled a high Q-factor and resonance frequency of 7748 MHz and 1.402 MHz, respectively, to be achieved even in liquids, facilitating the analysis of human salivary cortisol. The line-free mass sensor could be used for repeated measurements with the microfluidic mechanism, and the resonator could be fully washed out. It was concluded that the microfluidic line-free mass sensor was suitable to analyze the concentration of a salivary hormone, cortisol, in human saliva samples, and that it provided high-throughput suitable for point-of-care testing.

## 1. Introduction

Building on near-patient monitoring, numerous biosensors have been introduced for the diagnosis of diseases and for health monitoring [1]. The growing demand for compact biosensor devices for analyzing human samples such as proteins, mRNA, and DNA on the micro- or nano-liter scale eventually led to the development of the lab-on-a-chip concept and micro total analysis systems [2,3]. Point-of-care testing (POCT) is particularly valued for the portability, immediate availability of test results, and multiple monitoring capabilities that it provides [4]. Additionally, supplying a saliva sample evokes less anxiety for patients than providing a blood sample, and less embarrassment than producing a urine specimen. Unlike the phlebotomy skills required for blood collection, saliva samples are easily procured. Multiple sampling over a day or over many days can be readily completed in the field or at home, thus increasing the feasibility of longitudinal studies [5]. Thus, noninvasive measurement techniques using saliva are suitable for POCT. The intersection of the growing body of knowledge about mental health bioindicators with advances in proteomic technologies represents great potential for the early detection, diagnosis, and management of a range of stress-related disorders. Of particular interest is the stress hormone cortisol, which reflects the underlying neuroendocrine response to stressors and is commonly used as marker of stress reactivity [6,7]. However, it is necessary to increase the sensitivity by about 10 times for analyzing salivary biomarkers compared with blood analysis [8]. The proportion of salivary cortisol to total cortisol is about 1–2% in the lower range, but about 8–9% in the upper range.

Various novel detection techniques for biomarkers have been proposed to enable improved sensitivity, sample preparation, chemical manipulation and reaction, high-throughput, and portability [9]. The assay type of biosensor can be classified into direct, competitive, label-free, and enzymatic techniques [10]. The label-free technique for target particles satisfies the demand not only for reusability of the sensor, but also for miniaturization and portability of the sensor system [11]. Based on this technique, label-free immunosensors for highly sensitive detection of biomarkers have been reported [12,13,14].

Microcantilever biosensors undergo bending if the molecular adsorption is confined to a single surface [15,16]. Microcantilever biosensors are suitable for use with the label-free technique, because the frequency shifts sensitively in response to mass loading from molecular interactions. Sauerbrey developed a method for conversion of frequency to mass using quartz crystals, which is valid in nearly all applications [17,18]. The frequency shift is proportional to the glued-on mass. Sauerbrey’s equation defined that the frequency change is proportional to the square of the initial resonance frequency. With regard to theoretical study, Lavrik et al. revealed that the potential measurement sensitivity of microcantilever biosensors is 2.34 × 10^−19^ g [19]. Microcantilever biosensors have attracted much attention owing to their potential as a platform for the development of a myriad of physical, chemical, and biological sensors [20,21,22]. However, when the excitation source is directly fixed to the microcantilever, the electrical power required to feed it seriously degrades the vibrations and, consequently, decreases the quality factor (Q-factor) of the microcantilever biosensor. To address this, microcantilever biosensors have been proposed in which the sensing element—i.e., the resonator—is separate from the excitation source. Teva et al. reported an electrostatically driven resonant cantilever used as a resolution mass sensor [23]. However, very precise positioning of the microcantilever to maintain the gap between the microcantilever and the excitation source is required.

I have previously proposed a novel strategy in which the excitation source and the mass sensing element are separated. This mechanical resonance type biosensor was described as a line-free mass sensor comprising a resonator and an excitation source separate from the resonator, so that no electrical connections were required to be attached to the resonator [24]. This strategy has two advantages: (1) the Q-factor remains high, because the resonator is separate from the excitation source; and (2) the whole body of the resonator can be washed out completely, because the resonator exists separately, making this structure suitable for use in label-free techniques. The theoretical mechanism and tentative measured results of a line-free mass sensor were described using a simple beam-shaped resonator of AT-cut crystal at a resonance frequency of 145.5 kHz, but the sensitivity was insufficient (≅76.2 ng/mL) [25].

This study aims to describe the contribution of a line-free mass sensor that can achieve enough sensitivity for salivary cortisol analysis in a sub ng/mL range, as well as a high-throughput function suitable for POCT, by applying a microfluidic mechanism and label-free technique. A new mechanical resonator for a line-free mass sensor was designed and fabricated by photolithography to improve the Q-factor and thus increase the resonance frequency in the MHz range. A microfluidic platform was designed to enable washing of the detection area to improve the sensitivity. A label-free technique for capturing and releasing the target material from an antibody used in the immunoreaction was applied to achieve reusability and high-throughput.

## 2. Materials and Methods

### 2.1. Chemicals

Sulfuric acid (H_2_SO_4_; CAS No. 7664-93-9, Wako Pure Chemical Industries Ltd., Osaka, Japan) and hydrogen peroxide (30% H_2_O_2_; CAS No. 8007-30-5, Wako Pure Chemical Industries Ltd.) were used for preparing the piranha solution. A 10-carboxydecylphosphonic acid (10CDPA, Dojin Chemistry Laboratory Ltd., Kumamoto, Japan) was used to form a self-assembled monolayer (SAM) membrane. N-hydroxysulfosuccinimide (CAS No. 6066-82-6, Thermo Scientific Pierce Protein Research Products, Thermo Fisher Scientific, Waltham, MA, USA) and 1-ethy1-3-carbodiimide (CAS No. 1892-57-5, amine coupling kit A515, Dojin Chemistry Laboratory Ltd.) were used to activate the SAM membrane, and ethanolamine (amine coupling kit A515, Dojin Chemistry Laboratory Ltd.) was used to deactivate it. A monoclonal anti-cortisol antibody (2330-4879, host: mouse, AbD Serotec, Kidlington, Oxfordshire, UK) was used for the immunoassay. A phosphoric acid buffer solution (PBS; pH 7.3, 1 mM, Dulbecco A, Oxoid Ltd., Hampshire, UK) was used as a buffer solution. Glycine (N_2_NCH_2_COOH; CAS No. 56-40-6, 0.1 M, Wako Pure Chemical Industries Ltd.) and hydrochloric acid (HCl; CAS No. 7647-01-0, 0.1 M, Wako Pure Chemical Industries Ltd.) were used for preparing the dissociation solution. Cortisol-3-bovine serum albumin (Coltisol-3-CMO-BSA, Cosmo Bio Co., Ltd., Tokyo, Japan) was used to prepare a cortisol sample solution. A cortisol enzyme-linked immunosorbent assay (ELISA) kit (1-3002, monoclonal antibody to cortisol, 450 nm measurement wavelength, Salimetrics LLC, Carlsbad, CA, USA) was used to determine the calibration curve. The analysis was carried out in accordance with a protocol provided by the manufacturer [26]. BSA (CAS No. 9048-46-8, Wako Pure Chemical Industries Ltd.) was used to prepare a model saliva with similar total protein concentration to that in humans, which ranges between 1 g/L and 3 g/L [27].

### 2.2. Structure and Principle

**Figure 1:**
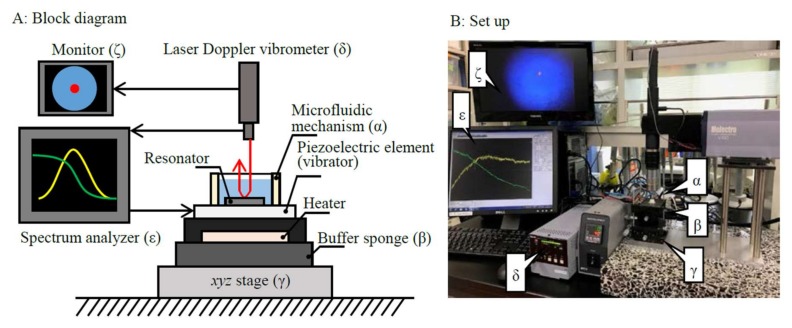
Block diagram of a line-free mass sensor and the resonance frequency measuring system. Electrical power is used to actuate the piezoelectric element, leaving the line-free mass sensor free from power lines.

A line-free mass sensor consists of a disk-shaped mechanical resonator (disk-shaped resonator), a separate piezoelectric element (20 mm diameter and 0.5 mm thickness, NEC Tokin Corporation, Miyagi, Japan) used to excite vibrations in the resonator (vibrator), and a fabricated microfluidic mechanism (Figure 1A,B). For use as a mass sensor, the line-free mass sensor is placed on an *xyz*-stage (TSD-805S, Sigmakoki Co., Ltd., Tokyo, Japan) across a buffer sponge (thickness of 10 mm). A heater is used to maintain the temperature of the fluid (MPHK 50*50, Misumi Group Inc., Tokyo Japan). A spectrum analyzer is used as an alternating current power source (R3755A, Advantest Corporation, Tokyo, Japan). Since the power source is used to actuate the vibrator, the sensing element (i.e., the disk-shaped resonator) is free from power lines. A laser Doppler vibrometer microscope (KV-100, Denshigiken Corporation, Kanagawa, Japan) and a spectrum analyzer (1 mHz frequency resolution) were used for measuring the resonance frequency of the disk-shaped resonator.

**Figure 2:**
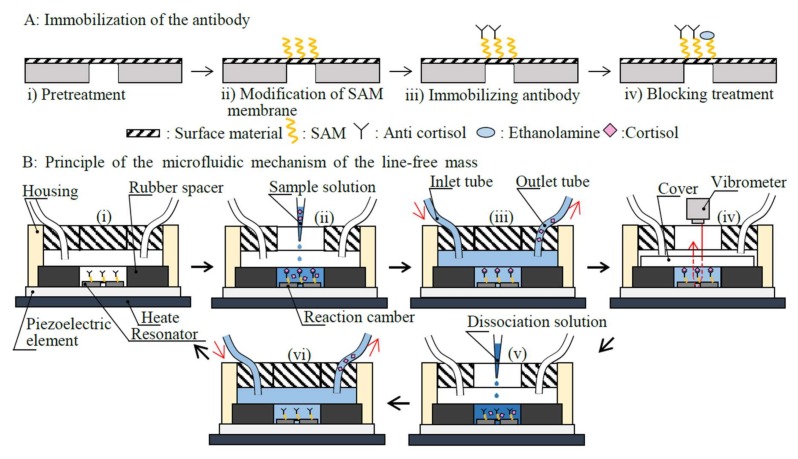
Schematic of immobilization of the antibody and the principle of the line-free mass sensor using the microfluidic mechanism (the schematic is not drawn to scale).

The procedure used to apply a label-free technique for the disk-shaped resonator was as follows (Figure 2A).

Step i: As a pretreatment, the surface material (aluminum) of the disk-shaped resonator was cleaned of organic residue using piranha solution for 10 min. Nitrogen gas was sprayed onto the flat electrode to dry it.
Step ii: A 10-carboxydecylphosphonic acid was used to form the SAM membrane on the disk-shaped resonator. The disk-shaped resonator was soaked in a solution consisting of 100 mmol/L N-hydroxysulfosuccinimide and 100 mmol/L 1-ethy1-3-carbodiimide at 25 °C for 10 min to activate the SAM membrane.
Step iii: Subsequently, an anti-cortisol antibody (mass: *x* µg) was coupled to the SAM membrane using acetate buffer (10 mM, pH 5.5) for 30 min.
Step iv: Un-coupled activated carboxymethyl dextran was blocked with ethanolamine.

Figure 2B shows the principles of the microfluidic mechanism of the line-free mass sensor. The fabricated microfluidic mechanism (Figure 2(Bi)) comprised a rubber spacer (20 mm inner diameter), a 200 µL inner volume of housing (equivalent to the sample volume), an inlet tube (2 mm inner diameter), an outlet tube (2 mm inner diameter), a twin channel type rotary-pump (403U/VM, Watson-Marlow Bredel Pumps, Wilmington, MA, USA), and a cover (quartz glass Fujirika Kogyo Co. Ltd., Osaka, Japan). The inner volume (20 µL) of the rubber spacer was used as a reaction chamber.

First, a 200 µL of sample solution (saliva) is dropped into the housing using a micropipette (100–1000 µL, Eppendorf AG, Hamburg, Germany) and allowed to react at an appropriate microfluidic temperature (*T* °C) for 1 min to enable an immunoreaction to take place (Figure 2(Bii)). A washing buffer is introduced into the housing at a rate of 10 mL/min for an appropriate time; thus, a volume of washing buffer (*V* mL) is added through both the inlet and outlet tubes for washing out the remaining residues (Figure 2(Biii)). Afterwards, the washing buffer is removed from the housing and the cover is closed, with 20 µL of solution left in the reaction chamber (Figure 2(Biv)). When the piezoelectric element is excited at an arbitrary frequency, vibrations are transmitted to the disk-shaped resonator, which vibrates at its resonance frequency. The resonance frequency is measured by a laser Doppler vibrometer and is inversely proportioned to the mass of the target material captured by the disk-shaped resonator. After the measurements, the analyte, cortisol, is dissociated from the anti-cortisol antibody in order to enable its reuse by reacting with a dissociation solution (20 µL, pH 3.0) for the minimum dissociation time (*t* min: the time between the addition of the dissociation solution and the initiation of washing, Figure 2(Bv)). Finally, the washing procedure is repeated for the same condition as in Figure 2(Biii,Bvi). If needed, the line-free mass sensor can be used for a repeat measurement immediately after this procedure.

### 2.3. Design and Fabrication of Mechanical Resonator

**Figure 3:**
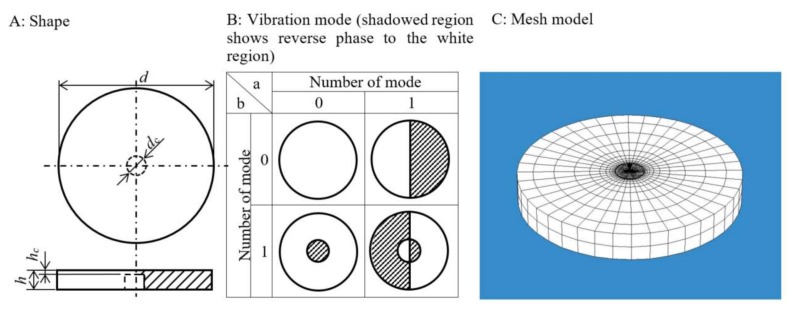
Proposed disk-shaped resonator.

A disk-shaped resonator with a thin plate portion at its center was proposed (Figure 3A). The center of the disk-shaped resonator was designed to vibrate as a peripheral fixed boundary condition in 0–1 mode (Figure 3B). Since the amplitude of the ultrasonic vibration was quite small, the resonator did not slip down from the piezoelectric element. This sensor used a standing wave, so the resonator would not move. In the theoretical equation of a disk-shaped resonator, the resonance frequency is proportional to the thickness of the center (*h*_c_) and inversely proportional to the square of the center diameter (*d*_c_) [28]. The outer-diameter (*d*), center-diameter (*d*_c_), thickness (*h*), and thickness of center (*h*_c_) of the disk-shaped resonator were determined by calculation using an eigenvalue–eigenvector evaluation employing a finite element method (COMSOL Multiphysics Ver. 5.20, COMSOL AB, Stockholm, Sweden) [29]. The total numbers of elements and nodes were 12,000 and 63,700, respectively (Figure 3C). The boundary conditions at the outer peripheral edge of the disk-shaped resonator were set to the free-free edge type. Si was selected as the material for the disk-shaped resonator, and the density, longitudinal modulus of elasticity, and Poisson’s ratio were set to be 2329 kg/m^3^, 185 GPa, and 0.28, respectively. Air (1.29 kg/m^3^ of density) and water (999.97 kg/m^3^ of density) were used as the media.

**Figure 4:**
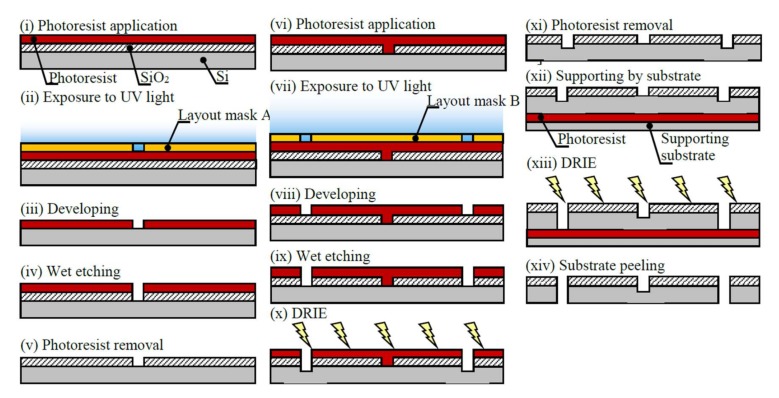
Photolithography with deep-etching of the disk-shaped resonator, including a two-step exposure process using layout masks A and B.

Figure 4 shows the manufacturing process of the disk-shaped resonator using photolithography with deep-etching. The wafer was coated with a positive-type photoresist (OFPR-800, Tokyo Ohka Kogyo Co., Ltd., Kanagawa, Japan) using a spin coater (ASC-4000, Actes Inc., Atsugi, Japan). The photoresist was exposed to a pattern for 8 s at an intensity of 48.0 mW/cm^2^ using layout mask A and a mask aligner (MA6, Suss MicroTec KK, Kanagawa, Japan). The silicon was wet (liquid)-etched to remove the unprotected oxide. The photoresist was removed from the substrate using a resist stripper.

The second photoresist layer was exposed for 24 s at an intensity of 48.0 mW/cm^2^ using layout mask B. Deep reactive ion etching (DRIE) was performed for 23 min using a Si DRIE system (MUC-21, Sumitomo Precision Co., Ltd., Osaka, Japan). The photoresist was removed using acetone, and the SiO_2_ was removed using hydrogen fluoride. Prior to the second DRIE for 92 min, a substrate was attached to support the wafer. Finally, the substrate was peeled from the disk-shaped resonator.

### 2.4. Tuning of Sensor Parameters

As mentioned in the description of the principle of the line-free mass sensor (2.2), four parameters affect the sensitivity: the mass of the anti-cortisol antibody, the temperature of the reaction chamber, the volume of washing buffer, and the dissociation time. Throughout these experiments, the cortisol standard solution was used as the sample solution. The optimal values for these parameters were determined experimentally as follows.

I. Mass of anti-cortisol antibody (*x*): Three different masses of anti-cortisol antibody, 0.4, 2, 4, and 20 µg. (4, 20, 40, and 200 µg/mL × 100 µL) were used as the parameters. The concentration of cortisol standard solution, the sample volume, the temperature of reaction camber, volume of washing buffer, and dissociation time were set to 100 ng/mL, 20 µL, 25 °C, 50 mL, and 30 min, respectively.

II. Temperature of reaction chamber (*T*): Three different reaction chamber temperatures, 30, 40, and 50 °C, were used as the parameters. The concentration of cortisol standard solution, the sample volume, the mass of anti-cortisol antibody, the volume of washing buffer, and the dissociation time were set to 100 ng/mL, 200 µL, 4 µg, 50 mL, and 30 min, respectively.

III. Volume of washing buffer (*V*): Three different volumes of washing buffer, 20, 50, and 100 mL (2, 5, and 10 min × 10 mL/min), were used as the parameters. The concentration of cortisol standard solution, the sample volume, the mass of anti-cortisol antibody, the temperature of the reaction chamber, and the dissociation time were set to 20 µg/mL, 20 µL, 4 µg, 25 °C, and 30 min, respectively.

IV. Dissociation time (*t*): Three different dissociation times, 10, 30, and 60 min, were used as the parameters. The concentration of cortisol standard solution, the sample volume, the mass of anti-cortisol antibody, the temperature of the reaction chamber, and the volume of washing buffer were set to 20 µg/mL, 20 µL, 4 µg, 25 °C, and 50 mL, respectively.

The frequency shift, Δ*f*, of the primary bending mode of vibration due to the sample being applied to the disk-shaped resonator was measured.

Unless otherwise stated, the measurements were repeated three times (*n* = 3), and all data are expressed as the mean ± standard deviation (SD).

### 2.5. Calibration Curve

A feasibility study using the fabricated line-free mass sensor was conducted to measure the concentration of cortisol using both cortisol standards and native human saliva, with a 200 µL sample volume. The four parameters, mass of anti-cortisol antibody, temperature of reaction chamber, volume of washing buffer, and dissociation time, were 20 µg, 40 °C, 50 mL, and 30 min, respectively.

A set of cortisol standards ranging from 0.1 ng/mL to 10 ng/mL was used. Distilled water and a concentration of protein solution (model saliva) were used as the solvent for the sample solutions. The concentration of protein in the model saliva was set to 2 g/L, which was similar to the total protein concentrations in saliva [27]. For the evaluation, the coefficient of determination (R^2^), the coefficient of variation (CV), and the limit of detection (LoD) using a blank sample were calculated.

The whole saliva samples were obtained from seven healthy male young subjects (24.0 ± 0.5 year) using collection procedures approved by the Ethics Committee of Shinshu University. A passive drool saliva sample (≅500 µL) was collected individually from the subjects. Each subject provided the sample by allowing saliva to pool at the bottom of the mouth and then expectorating the saliva into a commercially available sample tube (LT-0200, Ina-optika Co., Ltd., Osaka, Japan). The whole saliva was centrifuged using a filter (Ultrafree-MC, Nihon-millipore Co., Ltd., Tokyo, Japan) for 30 min (11,000× *g* = 85 mm × 10,800 rpm 24 °C). The volume of the saliva sample was set to 200 µL for each cortisol analysis. The measurements were repeated five times (*n* = 5).

The salivary cortisol levels were measured using cortisol ELISA kits and a plate reader (ARVO MX; Perkin Elmer Life Science, Boston, MA, USA) as a conventional assay. A set of human saliva samples between 1.64 ng/mL and 4.72 ng/mL were used to establish calibration curves for the line-free mass sensor.

## 3. Results and Discussion

### 3.1. Design and Fabrication of Mechanical Resonator

**Figure 5:**
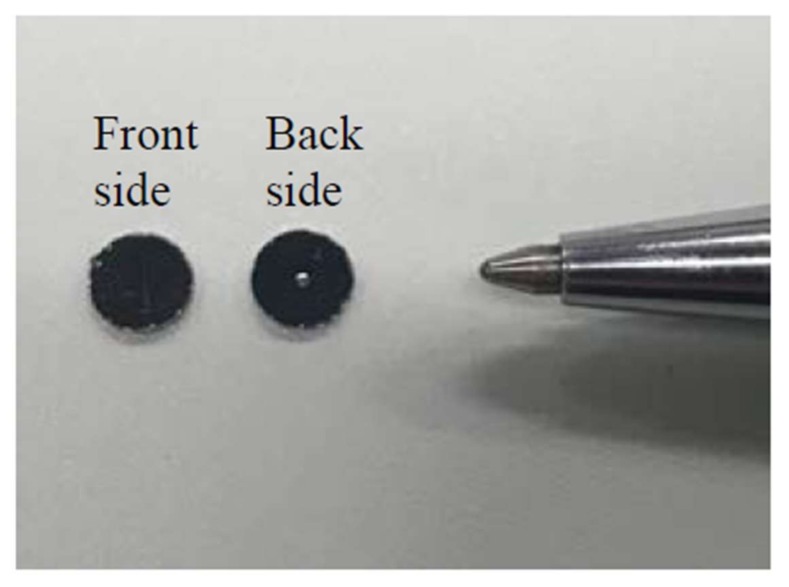
Disk-shaped resonator fabricated by photolithography (*d* = 4 mm, *d*_c_ = 0.5 mm, *h* = 0.5 mm, *h*_c_ = 0.1 mm).

**Figure 6:**
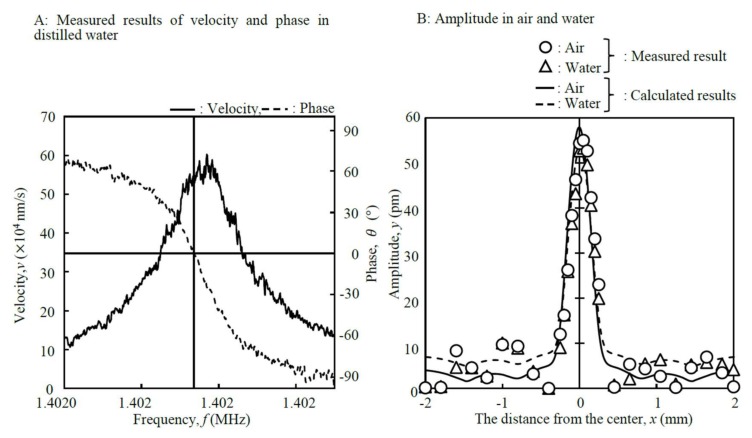
Resonance characteristics of the fabricated disk-shaped resonator at room temperature (25 °C).

Figure 5 shows the fabricated disk-shaped resonator imaged by photolithography. The dimensions were *d* = 4 mm, *d*_c_ = 0.5 mm, *h* = 0.5 mm, and *h*_c_ = 0.1 mm, respectively. Table 1 shows the calculated and measured results for the resonance frequencies of the disk-shaped resonator for each medium. The measurements were performed at room temperature (25 °C). In order to obtain high sensitivity, the dimensions of the disk-shaped resonator were selected so that the resonance frequency would be 4 MHz in the air. The calculated result was 3.862 MHz, which agreed well with the measured result of 3.841 MHz. The resonance frequency of the disk-shaped resonator decreased to 1.402 MHz when it was dipped in distilled water. The Q-factor calculated from the measured result of the velocity in distilled water was 7748 (Figure 6A). The Q-factor was comparatively high compared with those given in previous reports of between 3390 and 6800 [30,31]. The measured amplitude of the disk-shaped resonator agreed well with the calculated value in both media (Figure 6B). Based on the results, the vibrating area was estimated to be 0.20 mm^2^.

### 3.2. Tuning of Sensor Parameters

**Figure 7:**
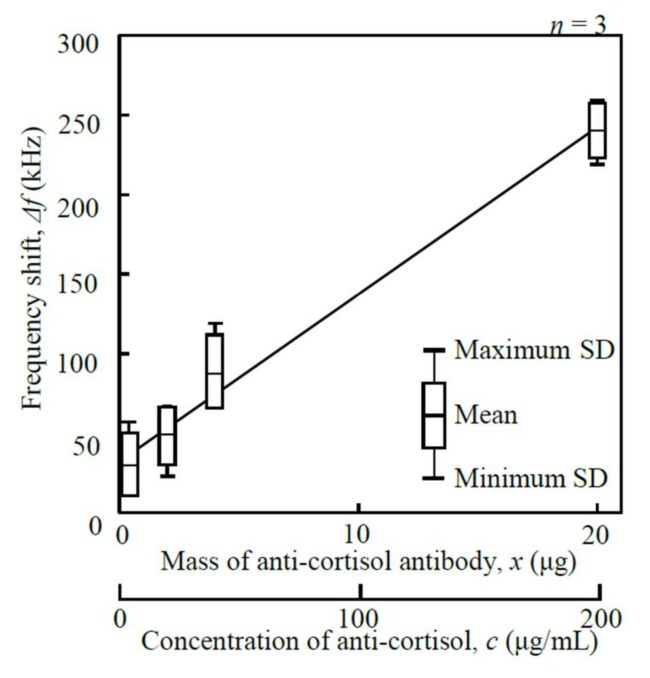
Relationship between the frequency shift, Δ*f*, and the mass of anti-cortisol antibody, *x* (1.402 MHz measured frequency).

I. Mass of anti-cortisol antibody (*x*): Figure 7 shows the relationship between the frequency shift, Δ*f*, and the mass of the anti-cortisol antibody, *x*, measured at 1.402 MHz in a range between 0.4 µg and 20 µg. The frequency shift increased proportional to the mass of anti-cortisol antibody immobilized on the surface of the disk-shaped resonator, and Δ*f* reached its maximum of 240.0 ± 16.4 Hz. From a cost perspective, 20 µg was selected.

**Figure 8:**
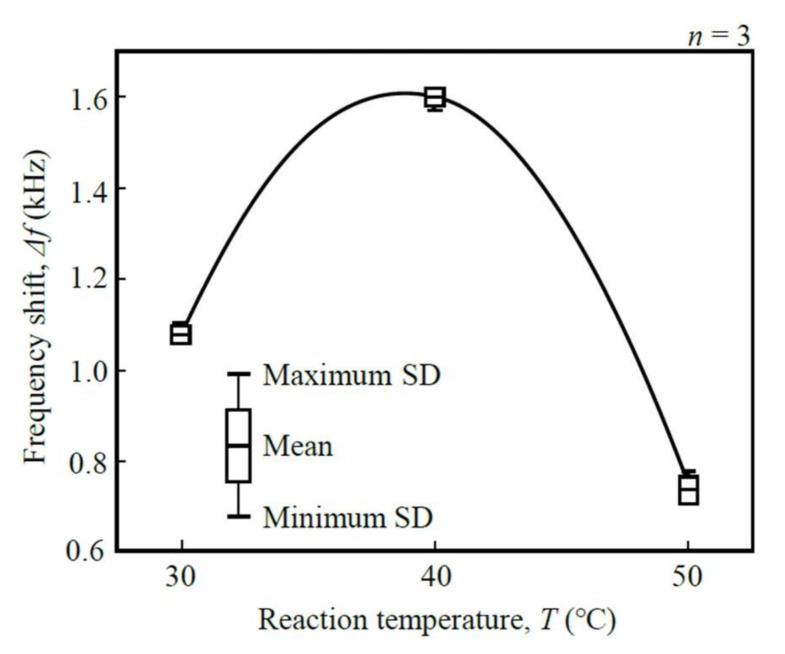
Influence of the temperature of reaction chamber, *T*, on the frequency shift, Δ*f* (1.402 MHz measured frequency).

II. Temperature of reaction chamber (*T*): The influence of the temperature of the reaction chamber, *T*, on the frequency shift, Δ*f*, was evaluated under conditions around human body temperature (Figure 8). The frequency shift reached its maximum of 1599.3 ± 21.3 Hz when the temperature of the reaction chamber was 40 °C. It was concluded that 40 °C should be used for the immunoreaction of the anti-cortisol antibody.

**Figure 9:**
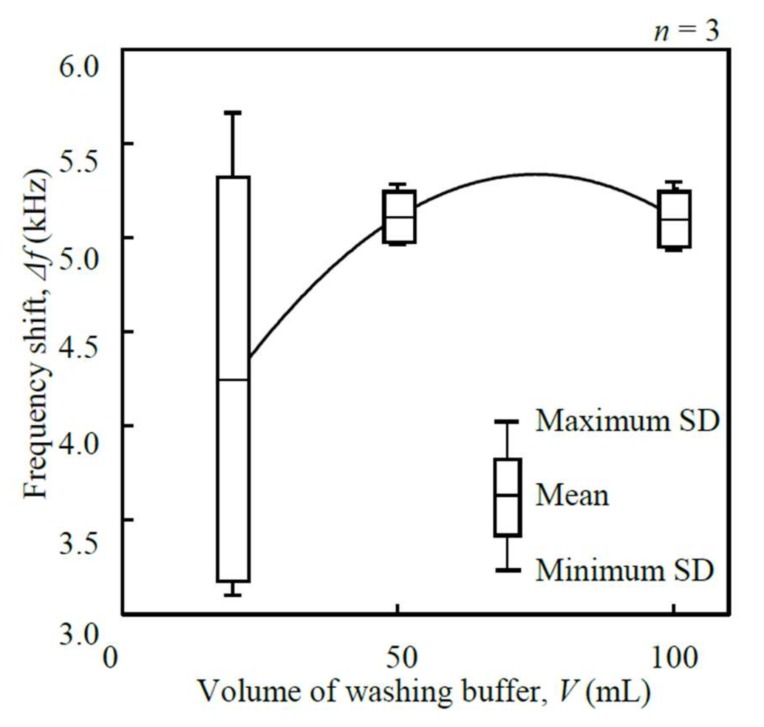
Relationship between the frequency shift, Δ*f*, and the volume of washing buffer, *V* (1.402 MHz measured frequency).

III. Volume of washing buffer (*V*): The volume of washing buffer, *V*, was optimized in order to maximize the frequency shift, Δ*f* (Figure 9). The flow rate was set to be constant at 10 mL/min, because too high a flow rate would cause peel-off of the antibody. The washing procedure was considered necessary because the SD at 20 mL was very large. The frequency shift became saturated as the volume of washing buffer was increased. The frequency shift reached a maximum of 5110.0 ± 132.4 Hz when the volume of washing buffer was 50 mL. Thus, it was concluded that 10 mL/min × 5 min was enough to remove impurities from the reaction chamber of the microfluidic mechanism.

**Figure 10:**
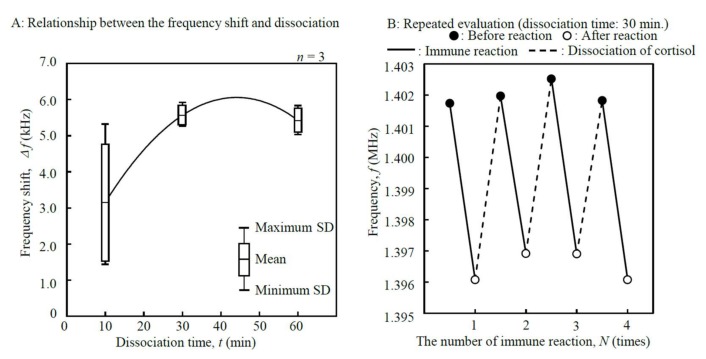
Results of repeated evaluation when immunoreaction and dissociation were performed using one disk-shaped resonator (1.402 MHz measured frequency).

IV. Dissociation time (*t*): If the dissociation time, *t*, is insufficient, the frequency shift will decrease. The dissociation time was optimized not only to maximize the frequency shift, Δ*f*, but also to enable reuse of the immobilized anti-cortisol antibody for sensing (Figure 10A). The frequency shift reached its maximum of 5564.0 ± 276.0 Hz when the dissociation time was 30 min. Figure 10B shows the repeated evaluation when both immunoreaction and dissociation were performed using one disk-shaped resonator with 30 min of dissociation time. The CV of Δ*f* in the immunoreaction was 4.96% when the measurements were repeated *N* = 4 times. One report describes more than 100 cycles of repeated measurements with a label-free technique, the binding was 96% of the initial binding [32]. Similar numbers of repeated measurements might be possible to be a motif with other label-free techniques.

### 3.3. Calibration Curve

**Figure 11:**
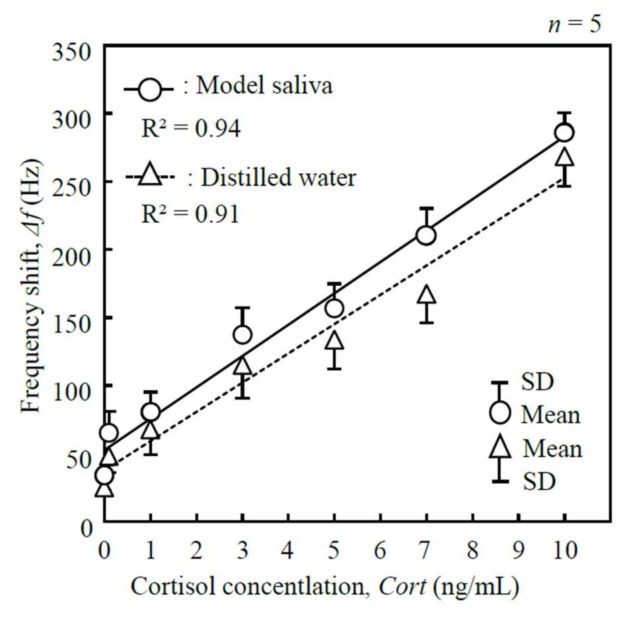
Calibration curves of standard cortisol samples in distilled water and model saliva as solvents (1.402 MHz measured frequency, 200 μL sample solution).

Calibration curves for the line-free mass sensor were established using a set of cortisol standards ranging between 0.1 ng/mL and 10 ng/mL (Figure 11). The results of a linear regression analysis for distilled water showed an R^2^ value of 0.91, CV of 24.2%, and LoD of 0.879 ng/mL; the relationship between the frequency shift, Δ*f*, and the cortisol concentration, *Cort*, was given by *Cort* = 4.65 × 10^−2^ Δ*f* − 1.75 (ng/mL). The results of a linear regression analysis for model saliva showed an R^2^ value of 0.94, CV of 20.5%, and LoD of 1.094 ng/mL; the relationship between the frequency shift, Δ*f*, and the cortisol concentration, *Cort*, was given by *Cort* = 4.34 × 10^−2^ Δ*f* − 2.28 (ng/mL). The 0.879 ng/mL of LoD was in the sub-nano-grams-per-milliliter range.

### 3.4. Analysis of Human Salivary Cortisol

**Figure 12:**
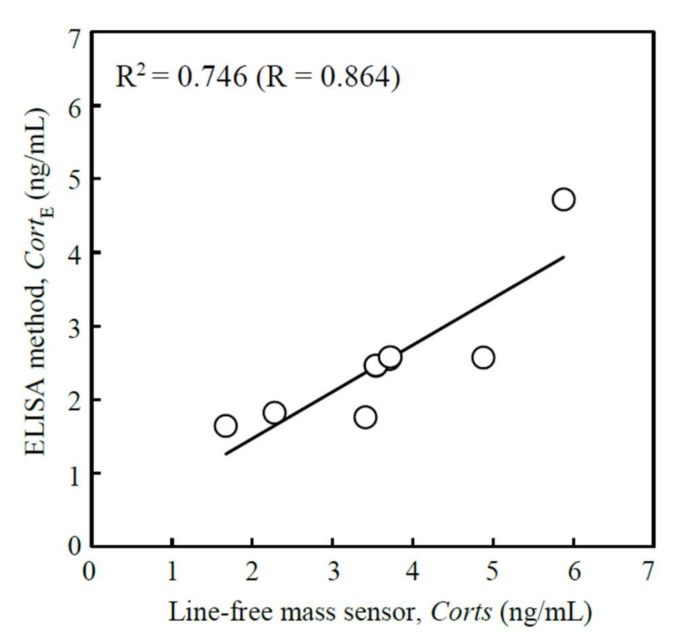
Scatter plot visualizing correlation of cortisol concentrations between a conventional assay (enzyme-linked immunosorbent assay (ELISA) method) and the fabricated line-free mass sensor in the use of human saliva samples.

The relationships between the conventional assay, the enzyme-linked immunosorbent assay (ELISA) method, *Cort*_E_, and the fabricated line-free mass sensor, *Cort*_S_, in the use of human saliva samples are shown graphically in Figure 12. The relationship was given by *Cort*_E_ = 0.6361 *Cort*_S_ + 0.1976 (ng/mL). The correlation coefficient, R^2^, was 0.746. The sensitivity of the line-free mass sensor was enough to analyze the human salivary cortisol when a 200 µL of sample saliva was used.

In the ELISA method, the total process time is more than 4 h or half a day. Additionally, to use the ELISA method, experience of chemical analysis is necessary. The label-free technique was successfully applied to the disk-shaped resonator using 50 mL of washing buffer which enabled several repeated measurements [33]. The total process time was <50 min, including both washing processes (each for 5 min) and 30 min of dissociation time to enable reuse of the line-free mass sensor.

It was concluded that salivary cortisol can be analyzed for a sensitivity of 1 ng/mL using the microfluidic line-free mass sensor. Saliva is attracting increasing attention as a biofluid that can be used as a source of biological indicators, not just of oral diseases [34] but also of general systemic disease precipitated by stress [35,36]. Analysis of salivary hormones such as cortisol would be medically useful.

## 4. Conclusions

A microfluidic line-free mass sensor for salivary cortisol as a biomarker was described that enables both high sensitivity and high-throughput. The disk-shaped resonator and the microfluidic mechanism were designed to enable all of the required functions, such as temperature control, washing of the reaction chamber, and a dissociation process for the analyte. The line-free mass sensor showed both a high Q-factor and high resonance frequency, even in liquid, of 7748 and 1.402 MHz, respectively, enabling the analysis of human salivary cortisol.

The line-free mass sensor enabled both repeated measurements with the microfluidic mechanism and a resonator that could be fully washed. Including the dissociation process needed for refreshment of the antibody used for molecular recognition of a target material, the measurement cycle would be less than 50 min, which represents a suitable time for an annual dental checkup.

The same line-free mass sensor can be used not only for the analysis of cortisol, but also in many other immunoassay applications by simply replacing the capture antibodies on the resonator. The microfluidic line-free mass sensor achieved high sensitivity in the sub-nano-grams-per-milliliter range, more improvement in sensitivity is desired in order to cover another applications.

## Figures and Tables

**Table 1:** Calculated and measured results of resonance frequencies of the disk-shaped resonator

Medium	Frequency (MHz)		Maximum Amplitude (pm)	Mesh Model
	Calculated	Measured		
Air	3.862	3.841	58	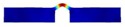
Distilled water	2.025	1.402	55	
